# Circulating Carnosine Dipeptidase 1 Associates with Weight Loss and Poor Prognosis in Gastrointestinal Cancer

**DOI:** 10.1371/journal.pone.0123566

**Published:** 2015-04-21

**Authors:** Peter Arner, Frauke Henjes, Jochen M. Schwenk, Spyros Darmanis, Ingrid Dahlman, Britt-Marie Iresjö, Peter Naredi, Thorhallur Agustsson, Kent Lundholm, Peter Nilsson, Mikael Rydén

**Affiliations:** 1 Department of Medicine (H7), Karolinska Institutet, Karolinska University Hospital, Huddinge, 141 86, Stockholm, Sweden; 2 Affinity Proteomics, Science for Life Laboratory, School of Biotechnology, Royal Institute of Technology, Box 1031, 171 21, Solna, Sweden; 3 Department of Surgery, Sahlgrenska Academy, University of Gothenburg, 413 45, Gothenburg, Sweden; 4 Division of Surgery, Department for Clinical Science, Intervention and Technology (CLINTEC), Södersjukhuset, 118 83, Stockholm, Sweden; GDC, GERMANY

## Abstract

**Background:**

Cancer cachexia (CC) is linked to poor prognosis. Although the mechanisms promoting this condition are not known, several circulating proteins have been proposed to contribute. We analyzed the plasma proteome in cancer subjects in order to identify factors associated with cachexia.

**Design/Subjects:**

Plasma was obtained from a screening cohort of 59 patients, newly diagnosed with suspected gastrointestinal cancer, with (n = 32) or without (n = 27) cachexia. Samples were subjected to proteomic profiling using 760 antibodies (targeting 698 individual proteins) from the Human Protein Atlas project. The main findings were validated in a cohort of 93 patients with verified and advanced pancreas cancer.

**Results:**

Only six proteins displayed differential plasma levels in the screening cohort. Among these, Carnosine Dipeptidase 1 (CNDP1) was confirmed by sandwich immunoassay to be lower in CC (p = 0.008). In both cohorts, low CNDP1 levels were associated with markers of poor prognosis including weight loss, malnutrition, lipid breakdown, low circulating albumin/IGF1 levels and poor quality of life. Eleven of the subjects in the discovery cohort were finally diagnosed with non-malignant disease but omitting these subjects from the analyses did not have any major influence on the results.

**Conclusions:**

In gastrointestinal cancer, reduced plasma levels of CNDP1 associate with signs of catabolism and poor outcome. These results, together with recently published data demonstrating lower circulating CNDP1 in subjects with glioblastoma and metastatic prostate cancer, suggest that CNDP1 may constitute a marker of aggressive cancer and CC.

## Introduction

Cachexia is a condition of involuntary weight loss observed in chronic disorders such as cancer, inflammatory diseases, congestive heart failure and end-stage renal disease [1]. As many as fifty percent of all cancer patients experience cancer cachexia (CC) which is strongly associated with reduced survival and poor response to antitumor treatment [2]. Reduced food intake may contribute to CC [3] but it does not solely explain its development, and nutritional supplementation fails to reverse the process [4]. Despite significant efforts, the underlying causal mechanisms remain unclear, and there is no effective treatment although several palliative strategies have been suggested [5,6,7,8].

Both fat mass and lean body mass are depleted in advanced CC [5,9]. However, longitudinal studies have demonstrated that loss of white adipose tissue (WAT) mass precedes that of muscle wasting [10,11]. Results in recent years have also shown that WAT mass reduction in CC is primarily associated with increased hydrolysis (lipolysis) [12] and oxidation [13] of adipocyte triglycerides. The mechanisms influencing lipid metabolism in CC are not known, but it is possible that factors released into the circulation, either directly from the tumour or indirectly from other tissues, may induce changes in fat cell lipolysis/lipid oxidation [12]. Several candidates have been proposed over the years, including zinc-alpha2-glycoprotein and tumor necrosis factor-alpha [14] although subsequent studies have shown that none of them can *per se* explain increased fat loss in CC [10,15].

Identification of novel circulating proteins associated with CC has been hampered for several reasons, not least the fact that plasma/serum is a complex matrix where the 20 most abundant proteins account for ~98% of the protein content [16]. Consequently, the detection of proteins present at lower levels requires affinity proteomic approaches where the major obstacle has been the availability of specific affinity reagents. Recently, several consortia have initiated efforts to solve this hurdle and produce specific affinity binders [17]. The Human Protein Atlas (HPA) project [18] aims at generating at least one antibody against each human protein and currently ~22.000 antibodies, targeting ~16.000 proteins, are represented with protein localisation and expression data on a publicly available database (www.proteinatlas.org).

We determined whether plasma from cancer patients with cachexia, in comparison to weight-stable cancer subjects (WS), display specific alterations in protein levels which associate with body weight/fat mass loss and/or other clinical features of CC. To this end, 760 antibodies (targeting 698 unique proteins) from the HPA Project were utilized on suspension bead arrays (SBA). This approach identified a small set of differentially expressed proteins among which Carnosine Dipeptidase 1 (CNDP1), a protein recently shown to be reduced in metastatic prostate cancer and glioblastoma [19,20], was confirmed by sandwich immunoassay to be reduced in CC. Circulating levels of CNDP1 were therefore compared with clinical measures of disease state.

## Materials and Methods

### Patients

The clinical characteristics of cohort 1 and 2 are detailed in Table 1. Cohort 1 consisted of 59 subjects with suspected gastrointestinal cancer, 43 males and 16 females, 26 of which have to a large extent been described elsewhere [21]. Thirty-two patients fulfilled the criteria of cachexia, i.e. involuntary weight loss exceeding five percent of habitual body weight in the preceding three months or ten percent in the preceding six months [21]. Twenty-seven reported no significant weight loss and were defined as weight-stable (WS). The CC patients were diagnosed with adenocarcinoma in the gastric area i.e. cardia/ventricle (n = 3), colon (n = 2 out of which one with liver metastases), pancreas (n = 11), oesophagus (n = 9, out of which 4 with squamous cell carcinoma) and cholangiocarcinoma (n = 1). The WS patients were diagnosed with adenocarcinoma in the oesophagus (n = 4), gastric area (n = 2), colon (n = 7, all with liver metastases), pancreas (n = 8) or cholangiocarcinoma (n = 1). Eleven subjects (six in the CC and five in the WS group), preoperatively determined to have a malignant disease, were postoperatively shown to have a non-tumour related diagnosis. In the CC group the final diagnosis was benign pancreatic cysts (n = 1), cholecystitis (n = 2), dysplasia in the oesophagus (n = 1) and chronic pancreatitis (n = 2). In the WS group the final diagnoses were cholecystitis (n = 1) and chronic pancreatitis (n = 4). As described in Results, although these subjects were included in the overall analysis, omission of these individuals did not have any major impact on the associations between CNDP1 and different clinical parameters. Cohort 2 consisted of 93 patients diagnosed with unresectable ductal pancreatic carcinoma. These samples were selected from a biobank collected during a previously described [22] palliative care program at the out-patient clinic of the Department of Surgery, Sahlgrenska University Hospital between 1993 and 2005. Only patients who were judged reasonably physically fit were included in the present study. None of the patients had signs of overt diabetes or any other additional cancer diagnosis or severe illness.

**Table 1:** Clinical characteristics of cohort 1 and 2.

	Cohort 1			Cohort 2
Parameter	CC (n = 32)	WS (n = 27)	p-value (CC vs WS)	
Gender (M/F)	26/6	17/10	0.12	52/41
Age (years)	64.2±8.8	63.7±8.5	0.83	69.9±10.9
BMI (kg/m^2^)	23.4±5.3	25.9±4.1	0.050	22.1±3.5
Lean body mass (kg)*	55.1±12	56.1±15	0.79	46.4±9.8
Fat mass (kg)*	16.1±14	22.0±11	0.074	15.4±8.5
Fat mass (%)*	20.4±13.0	28.2±11	0.021	24.4±9.1
% Weight loss	13.2±6.4	0.8±2.9	<0.0001	13.0±8.2
P-Glucose (mM)	6.5±1.6	6.0±1.4	0.16	7.4±3.2
P-Albumin (g/l)	34.9±3.7	37.8±2.9	0.0014	33.6±5.3
S-IGF (μg/l)	112±47	123±48	0.37	105±65
P-Transferrin (mM)	2.23±0.4	2.45±0.3	0.025	N/A
P-CRP (mg/l)	22.2±30	6.78±18	0.017	32±41
Leptin (ng/ml)	7.90±11	13.1±13	0.096	3.94±2.4
P-glycerol (μM)	106±60	88±37	0.20	50.7±31
P-glycerol/kg fat mass	11.8±12	4.68±3.1	0.0030	4.5±4.3
PG-SGA	9.8±5.2	2.0±1.3	<0.0001	N/A
RQ	0.81±0.04	0.84±0.06	0.020	0.79±0.06

Values are given as mean±S.D. except for gender where the actual number of males (M) and females (F) are given. Group values were compared using unpaired t-test or χ^2^ test.

*Measured by bioimpedance in cohort 1 and by DXA in cohort 2.

### Ethics Statement

Ethical approvals for the studies were obtained from the regional boards of ethics (in Stockholm and Gothenburg, respectively) prior to the start of the study, and all patients gave their signed informed consent. The study was performed in full accordance with the statements in the Declaration of Helsinki.

### Clinical examination

In cohort 1, the patients came to the laboratory after an overnight fast. Apart from measuring height and weight, body composition was determined by bioimpedance using a QuadScan 4000 (Bodystat Ltd, Isle of Man, British Isles). Indirect calorimetry to determine resting energy expenditure (REE) and respiratory quotient (RQ) was performed using Deltatrac (Datex-Engstroms, Helsinki, Finland). The nutritional status was assessed using a standardized questionnaire for oncology termed Patient-Generated Subjective Global Assessment (PG-SGA) [23]. A venous blood sample was used for the determination of circulating factors. In brief, 20 mL of venous blood was drawn from each donor and cold centrifuged to obtain plasma which was then aliquoted into 2 mL Eppendorf-tubes and stored at -80° C until use. Leptin levels were assessed by ELISA (RD systems, Abingdon, U.K.) while albumin, transferrin, insulin-like growth factor-1 (IGF1) and C-reactive protein (CRP) were determined by the accredited routine chemistry laboratory of the Karolinska University Hospital. Plasma glycerol was determined by bioluminescence as described [24] using heparin-plasma, samples were run in duplicates (2x25 μL) and mean values were corrected for total fat mass (in kg) as previously described [21]. The ratio of glycerol to total body fat weight was considered an index of *in vivo* lipolysis [21]. Subjects in cohort 2 were examined as described previously [11,22]. In brief, serum and plasma measurements were performed by the accredited routine chemistry laboratory of the Sahlgrenska University Hospital except for leptin which was determined inhouse by RIA (Linco Research Inc). Body composition was measured by dual-energy X-ray absorptiometry (DXA) using a LUNAR DPX-L scanner (Scanexport Medical, Helsingborg, Sweden). Whole body scans were obtained in fast scan mode. Body fat and lean tissue mass were analyzed using the extended research mode of the LUNAR DPX-L software (v1.31). REE and fat oxidation were measured by indirect calorimetry in the morning hours after an overnight fast using Deltatrac (Datex-Engstroms, Helsinki, Finland). The short form health survey questionnaire SF-36 (www.sf-36.org/tools/sf36.shtml) was used to measure quality of life according to physical functioning (PF), bodily pain (BP), role-physical (RP), general health (GH), the physical component summary (PCS), vitality (VT), social functioning (SF), role-emotional (RE), mental health (MH) and the mental component summary (MCS).

### Antibodies

All antibodies utilized in the initial screening and profiling originate from the resources of affinity reagents generated within the Human Protein Atlas (www.proteinatals.org). Protocols for antigen selection, cloning, expression, purification, and immunization of rabbits, followed by affinity purification to yield mono-specific polyclonal antibodies, and their characterization with western blots and antigen microarrays were applied as described previously [25]. All protein fragments used for immunization were produced with a His6-albumin-binding protein tag and a target protein part of 80–120 amino acids. The antibodies were compiled into sets of 380 for practical experimental reasons and two such sets were utilized. The inclusion criteria were only based on quality assurance and technical parameters such as positive validation on protein microarrays and within a broad panel of tissues in immunohistochemistry, as well as concentration of the antigen-purified polyclonal antibodies. Beyond that, no considerations were made regarding annotation or function of the targeted proteins. The final compilation was driven by which antibodies coincidentally were produced and validated at the time of selection.

### Antibody suspension bead arrays

Antibodies were coupled to color-coded magnetic beads (MagPlex microspheres, Luminex Corp.) according to the manufacturer’s protocol and as described previously [20]. The coupling efficiency for each antibody was determined via R-phycoerythrin-labeled anti-rabbit IgG antibody (Jackson ImmunoResearch Laboratories). A 384-plex bead mixture was created with 380 antigen-purified polyclonal HPA antibodies and four bead identities used as controls. The latter consisted of IgG from a non-immunized rabbit and antibody-free buffer as negative controls while anti-albumin and anti-human IgG antibodies (Dako) were regarded as positive controls. The mixture contained equal amounts of beads where each population of a distinct color-code was carrying a particular antibody. Plasma samples were labelled and analyzed in accordance with previous studies with minor changes. The sample plates (Abgene) were centrifuged (2 min at 2,000 rpm), and 3 μl of each sample was added to 22 μl of sterile-filtered PBS with a liquid handler (SELMA, Cybio). N-Hydroxysuccinimidyl ester of biotinoyl tetraoxapentadecanoic acid (Pierce) was then added at ten-fold molar excess to yield an overall 1:10 sample dilution followed by a 2-h incubation at 4°C in a microtiter plate shaker (Thermomixer, Eppendorf). The reaction was stopped by the addition of a 250-fold molar excess of Tris-HCl, pH 8.0 over biotin and incubated for another 20 min at room temperature (RT) prior to a final storage at -20°C. All samples were subsequently utilized without removing unincorporated biotin and diluted 1:50 in an assay buffer composed of 0.5% (w/v) polyvinyl alcohol and 0.8% (w/v) polyvinylpyrrolidone (Sigma) in 0.1% casein in PBS supplemented with 0.5 mg/ml rabbit IgG (Bethyl Laboratories). The samples were heat-treated in a thermocycler for 30 min at 56°C and 10 min at 23°C. Then, 45 μl was added to 5 μl of bead mixtures in a half-area flat-bottom 96-well plate (Greiner), and incubation took place overnight on an orbital shaker at 650rpm at RT. Beads were washed in wells with 3 x 100 μl of PBST (1 PBS, pH 7.4, 0.05% Tween20) on magnet using a plate washer (EL406, BioTek) followed by 10 min fixation with 50 μl 0.4% paraformaldehyde in PBS. Beads were washed again before 50 μl of 0.5 μg/ml R-phycoerythrin-labeled streptavidin (Invitrogen) in PBST was added and incubated for 20 min. Finally, beads were washed and measured in 100 μl of PBST using a dedicated instrument (FlexMap3D, Luminex Corp.).

### Sandwich Immunoassay

Bead-based sandwich immunoassays were conducted as previously described [26]. Two different bead assays were created, one containing several mono- and polyclonal antibodies targeting CNDP1 as well as control proteins [26] while the second contained one antibody (HPA008933) targeting CNDP1 as well as controls. In both cases, BAF2489 (R&D Systems) was used as detection antibody. Briefly, the plasma samples were diluted 1:500 in assay buffer and heat-treated in a thermocycler at 56°C for 30 min and 23°C for 10 min. Then, 45 μl of diluted sample were combined with 5 μl of the bead array in microtiter plates (Greiner), and incubation took place O/N on a shaker at RT and 650 rpm. Beads were washed on a magnet 3x with PBST using a plate washer (EL406, BioTek). This was followed by 1 h of 0.4 μg/ml biotinylated detection antibody, 3x washing with PBST, and 10 min with a solution containing 0.4% paraformaldehyde in PBS. Beads were washed again, and 0.5 μg/ml R-phycoerythrin-labeled streptavidin (Invitrogen) in PBST was added and incubated for 20 min. Finally, beads were washed and measured in PBST using a dedicated instrument (FlexMap3D, Luminex Corp.).

### Statistical methods

Continuous variables are presented as mean±SD in text and mean±SEM in figures. Group differences were determined by Wilcoxon-Rank-Sum-Test, Student’s paired or unpaired *t*-test as indicated. χ^2^ test was used for nominal variables. Correlations between continuous variables were performed using Spearman’s correlation (where normal distribution could not be demonstrated/assumed) or simple linear regression. Statistical significance was set at the level of *P*<0.05. In the discovery cohort, no power calculation was performed. Statistical computations were carried out using standard software packages.

## Results

### Clinical characteristics of the discovery cohort

In cohort 1, the CC and WS subjects were of similar age and gender distribution although both subgroups consisted predominantly of men (Table 1). As expected, CC patients reported more pronounced weight-loss and displayed signs of catabolism as evidenced by lower BMI, lower plasma albumin and transferrin levels as well as poorer nutritional status demonstrated by higher PG-SGA scores. The weight-loss observed in the cachectic group was primarily due to loss of fat mass, since lean body mass (LBM) was similar in the two groups. In concordance with previously published data, the cachectic subjects displayed lower respiratory quotient (RQ), an indirect measure of fatty acid oxidation, as well as signs of increased *in vivo* lipolysis evidenced by significantly elevated plasma glycerol levels (corrected for total fat mass). Taken together, the cachectic group in cohort 1 displayed primarily WAT mass loss as well as all the classical features and clinical hallmarks of disturbed lipid metabolism previously reported in CC.

### Affinity proteomic analysis of circulating factors

**Fig 1:**
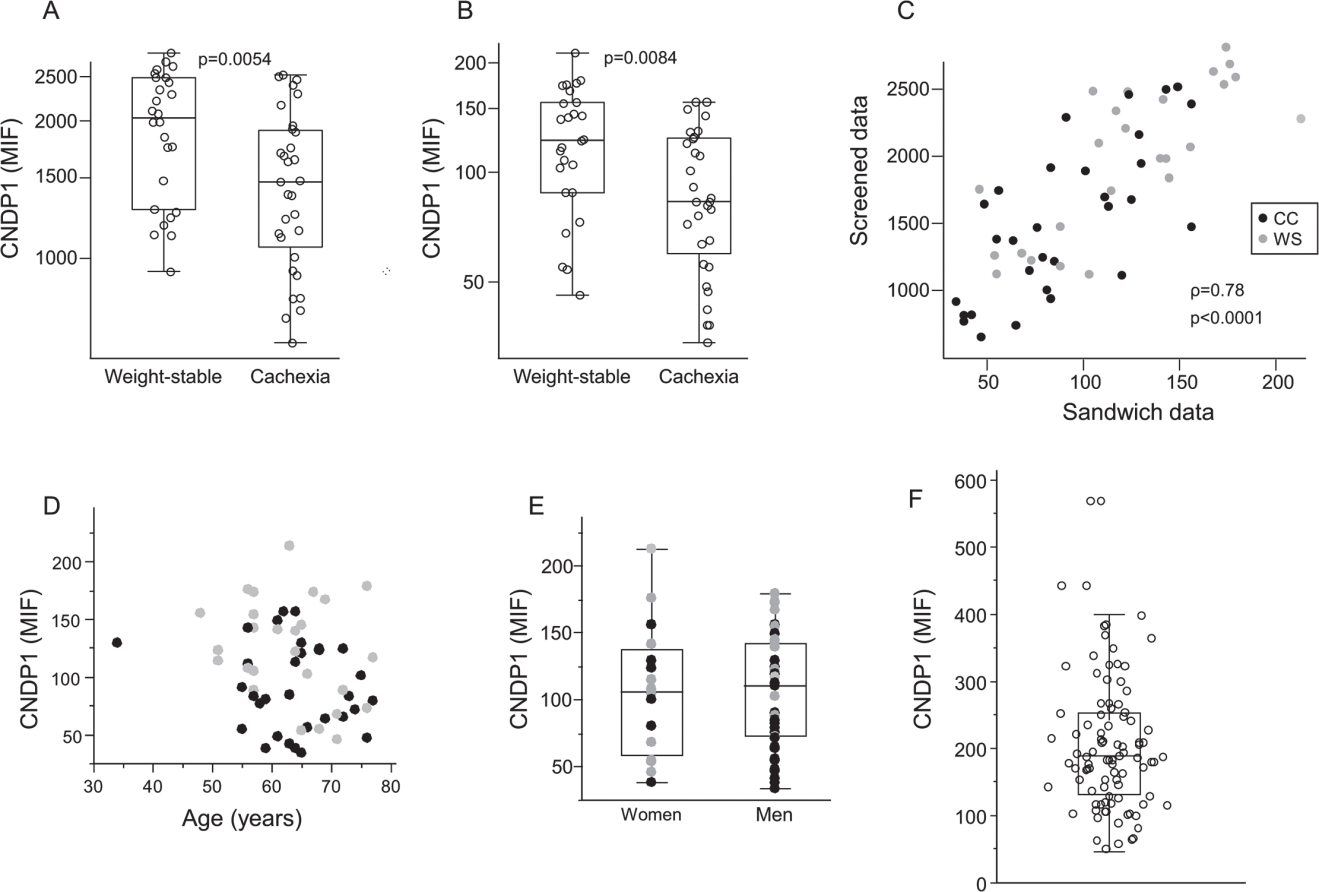
Identification and validation of CNPD1 reduction in CC. Detected protein levels are shown for **A.** the screen using Sandwich Bead Arrays (SBA) and **B.** validation by Sandwich Immunoassays (SIA) and expressed as mean intensity fluorescence (MIF). **C.** There was an excellent correlation between results obtained by SBA and SIA. CNPD1 levels measured by SIA were not influenced by **D.** age or **E.** gender. **F.** Distribution of circulating CNDP1 concentrations determined by SIA in Cohort 2. WS = Weight stable, CC = Cancer cachexia. P- and rho-values are given.

In order to identify circulating factors associated with CC, plasma samples from cohort 1 were analysed by proteomic profiling using suspension bead arrays (SBA, details on the antibody selection process are described in the Methods section). Out of the ~700 screened proteins, six showed significant differences between the CC and WS groups; three were lower; (CNPD1, APOA4, DACH1) while three were higher (BCL3 NARS2, ATP13A4) in CC subjects (Table 2). DACH1 is a transcription factor, BCL3 is a transcriptional co-activator, CNDP1, NARS2, ATP13A4 are suggested enzymes and APOA4 is a lipoprotein. CNDP1 and APOA4 are annotated as secreted extracellular factors while the remaining four are intracellular proteins. Furthermore, CNDP1 and APOA4 have been shown to be altered in plasma/serum of cancer subjects. Thus, reduced CNDP1 levels have been reported in different aggressive cancer forms [19,20] while APOA4 has been shown to be increased in pediatric forms of high risk acute leukemia [27] but lower in adult women with ovarian cancer [28,29]. Our studies were therefore focused on CNDP1 and APOA4 where the first step was to validate our SBA results by sandwich immunoassay (SIA). While the reduction in APOA4 could not be confirmed by SIA (S1 Fig, p = 0.21), CNDP1 was reduced in CC to a comparable degree in SBA (Fig 1A) and SIA (Fig 1B). Moreover, the individual CNDP1 values obtained using SBA or SIA were highly correlated (Fig 1C, ρ = 0.78, by Spearman’s rank correlation, p<0.001) and the reliability of the latter assay was further confirmed by two independent SIAs using different antibodies which revealed excellent intra- and inter-assay correlations (range of ρ = 0.93–0.99, graphs not shown). CNDP1 levels measured by SIA were not associated with either age or gender (Fig 1D and 1E, respectively).

**Table 2:** Candidates found in the unbiased proteomic screen.

Protein	Putative function (main GO annotations)	Gene ID	Serum levels in CC vs. WS	p-value
Carnosine Dipeptidase 1	metallopeptidase activity and dipeptidase activity	CNDP1	down	0.005
Apoliprotein A4	copper ion binding and protein homodimerization activity	APOA4	down	0.007
Dachshund Family Transcription Factor 1	RNA polymerase II core promoter sequence-specific DNA binding transcription factor activity involved in preinitiation complex assembly and nucleotide binding	DACH1	down	0.005
Asparaginyl-TRNA Synthetase 2	asparagine-tRNA ligase activity and nucleic acid binding.	NARS2	up	0.005
ATPase type 13A4	cation-transporting ATPase activity	ATP13A4	up	0.005
B-Cell CLL/Lymphoma 3	protein binding, bridging and sequence-specific DNA binding transcription factor activity	BCL3	up	0.004

### CNDP1 levels correlate negatively with factors associated with CC

Circulating CNDP1 levels were compared with different clinical parameters in cohort 1 (Table 3). There were positive relationships with BMI, fat mass (expressed in percent or absolute values) as well as with circulating levels of albumin, transferrin and IGF1. Conversely, CNPD1 was negatively associated with percent weight loss, PG-SGA score and *in vivo* lipolysis (expressed as P-glycerol/fat mass kg). The associations between CNDP1 and BMI, fat mass (kg), percent weight loss, serum-IGF1/transferrin and *in vivo* lipolysis remained statistically significant after exclusion of the eleven subjects that were diagnosed with non-malignant disease post-surgery.

**Table 3:** Correlation between CNDP1 and clinical parameters in cohort 1.

Parameter	r-value	p-value
BMI	0.41	0.0018
Lean body mass (kg)	0.055	0.69
Fat mass (kg)	0.46	0.0005
Fat mass (%)	0.41	0.0018
% Weight loss	-0.42	0.0012
P-Glucose	0.15	0.28
P-Albumin	0.35	0.0082
S-IGF1	0.42	0.012
P-Transferrin	0.43	0.0008
P-CRP	-0.16	0. 23
P-glycerol/total fat mass	-0.39	0.0031
PG-SGA	-0.30	0.025
RQ	0.23	0.088

Correlations were performed using simple linear regression, r- and p-values are shown.

### Reduced CNDP1 levels are associated with poor prognosis in a validation cohort

The SBA and SIA findings were obtained in a rather small cohort, diagnosed with different gastrointestinal cancers and where no outcome data is available. In order to validate but also to better define the association between reduced CNDP1 levels and prognosis, circulating CNDP1 was determined by SIA in a separate, more homogenous, validation cohort including only subjects with advanced pancreatic cancer (Cohort 2, Table 1 and Fig 1F). In these patients, CNDP1 values were negatively associated with percent weight loss and measures of fat oxidation while there was a positive relationship with plasma albumin, IGF1 and physical health-related quality of life measures (SF-36) (Table 4). Despite a lack of association between CNDP1 levels and established tumour markers (CEA, CA 125 and CA19-9), there was a significant positive correlation between CNDP1 and survival (measured as days of survival following diagnosis). In contrast to the findings in cohort 1, there was no association between CNDP1 and BMI, total fat mass or *in vivo* lipolysis.

**Table 4:** Correlations between CNDP1 and clinical parameters in cohort 2.

Parameter	r-value	p-value
BMI	-0.08	0.40
Fat mass (kg)	0.042	0.67
% Weight loss	-0.26	0.0038
P-Glucose	-0.11	0.27
P-Albumin	0.30	0.0006
S-IGF1	0.22	0.017
P-CRP	-0.19	0.032
P-CEA	0.14	0.37
P-CA 125	-0.20	0.20
P-CA 19–9	0.11	0.49
PF-SF36	0.29	0.0049
BP-SF36	0.33	0.017
RP-SF36	0.29	0.0062
PCS-SF36	0.34	0.0019
GH-SF36	-	0.99
VT-SF36	0.30	0.0039
SF-SF36	0.21	0.049
RE-SF36	0.18	0.09
MH-SF36	0.19	0.07
MCS-SF36	0.22	0.04
Fat oxidation (g/day)	-0.21	0.024
Survival (in days)	0.22	0.015

Correlations were performed using simple linear regression, r- and p-values are shown.

## Discussion

To our knowledge, the circulating proteome in CC has not been characterized before. Using our previously described antibody suspension bead array technology [30], we performed a screening of approximately 700 proteins. We found that only a limited set of factors, corresponding to less than 1% of the screened proteins, displayed significant differential plasma levels in CC-patients compared with weight-stable cancer subjects. This could possibly depend on the fact that the subjects in this screening cohort consisted of newly diagnosed cancer patients and were analysed at an early stage of CC or that indeed few circulating proteins associate with CC. It is also possible that we may have missed some relevant proteins due to the fact that many signals in a proteomic screen may be in the noise range and/or that the screen included antibodies directed against only a limited set of proteins. Among the differentially expressed proteins, four factors (CNDP1, APOA4, DACH1 and BCL3) have previously been studied in different aspects of cancer, but only APOA4 and CNDP1 constitute secreted proteins. The latter two have previously been studied in the circulation of cancer subjects, albeit not in the context of cachexia. Sandwich immunoassay confirmed lower plasma levels of CNDP1 in CC and low CNDP1 levels associated with several measures of CC. The main findings could be validated in a separate cohort demonstrating that reduced CNDP1 correlated with weight loss, more advanced disease states, and reduced survival. Admittedly, some of the associations differed between the two cohorts. Thus, while BMI, fat mass and *in vivo* lipolysis correlated with circulating CNDP1 in cohort 1, these relationships were not significant in Cohort 2. This could possibly depend on the fact that subjects in cohort 2 had a more advanced disease stage.

Eleven of the patients in cohort 1 were ultimately diagnosed with non-malignant disease. We do not regard this to have any major bearing on our results regarding CNDP1, partly based on the fact that omitting these individuals from the analyses did not have any major impact on the significant associations with different clinical parameters. More importantly, however, similar correlations with CNDP1 were found in cohort 2 which consisted only of subjects with confirmed pancreatic cancer.

The *CNDP1* gene encodes a 57 kDa glycoprotein of the M20 metallopeptidase family. In humans, it is primarily expressed in the central nervous system and (to a lesser extent) in the liver from where it is secreted as a homodimer. [31]. Although the sequence identity of *CNDP1* is highly conserved between species, the non-human gene is almost exclusively expressed in the kidney but because it lacks an N-terminal signal peptide it is not detectable in the circulation [31]. The function of CNDP1 is not fully defined but it displays carboxy-and dipeptidase activity and its main substrates appear to be the dipeptides carnosine (β-alanyl-histidine) and homocarnosine (γ-aminobutyryl-L-histidine) [31,32,33]. Carnosine is primarily present in muscle and brain tissue and has been implicated in multiple pathophysiological processes including a protective role in atheroscleroisis [34], hepatic steatosis [35], diabetic nephropathy [36,37,38,39] and in cancer [40], where its anti-proliferative properties on different malignant cells have spurred particular interest [41,42,43]. Low CNDP1 levels have been found to be associated with lymph node metastasis in a large cohort of prostate cancer patients [20]. Furthermore, a recent proteomic analysis using a non-immune-based (liquid chromatography-tandem mass spectrometry) approach suggested that CNDP1 is one of several proteins down regulated in plasma of ten patients with glioblastoma compared with healthy controls [19]. It is therefore possible that reduced CNDP1 levels may be a common denominator in several more aggressive cancer forms. Admittedly, it might seem counterintuitive that carnosine is proposed to have anti-tumour effects while reduced levels of a carnosine degrading enzyme associate with advanced cancer stages. However, it could be hypothesized that CNDP1 down regulation, resulting in increased carnosine levels, may constitute a compensatory/defensive mechanism activated in conditions with high cell turnover. Whether plasma carnosine levels are altered in cancer is not known, and we did not have sufficient material to determine the relationship between CNDP1 and carnosine in the circulation. Therefore, the unclear function of CNDP1 *in vivo*, as well as the design of our study, do not allow us to establish a causal link between reduced CNDP1 levels and CC.

The mechanisms controlling the circulating levels of CNDP1 are not known and we cannot exclude that the tissue/cellular source of CNDP1 may differ between healthy subjects and cancer patients. Furthermore, whether CNDP1 reduction occurs in other forms of weight loss is not known. As we have previously generated global transcriptomic data from WAT of cohort 1 [44], we examined whether *CNDP1* gene expression was affected by CC. The fact that no differences were observed (data not shown) suggests that the alterations in circulating CNDP1 observed in the present study cannot be explained by altered *CNDP1* expression in WAT. A more extensive mapping of CNDP1 expression in other conditions is necessary in order to elucidate how, and in which tissue, body weight loss primarily impacts on gene expression and protein secretion. A caveat mentioned above, which precludes mechanistic studies of CNDP1 in animal models, is that the murine orthologue is not secreted into the circulation. Indeed, although the SIA developed in the present work should theoretically recognize mouse CNDP1 (based on sequence homology) we were not able to observe any detectable levels in serum/plasma of several different mouse strains (data not shown).

CNDP1 undergoes posttranslational modifications including homodimerization and glycosylation. It is at present unclear how glycosylation status affects the activity and functional role of CNDP1. Although it may affect antibody affinity, the fact that CNDP1 levels correlated strongly between assays using four different antibodies, suggests that differences in glycosylation status are not likely to explain the differential levels observed in the CC and WS groups.

In conclusion, profiling of ~700 circulating proteins in gastrointestinal cancer shows that CNDP1 levels are reduced in cachexia and that low serum concentrations correlate with several CC parameters associated with poor outcome. The fact that only a limited set of plasma proteins are altered in CC suggests that few circulating factors are potential CC markers, at least in early phases of the disease. Whether CNDP1 has a prognostic value cannot be established in the present pilot study and needs to be validated in much larger cohorts. Furthermore, the primary tissue/cellular source, the mechanisms controlling its circulating levels as well as the functional role of CNDP1 remain unclear and will be the focus of future studies.

## Supporting Information

S1 Fig APOA4 levels in validation assay. Detected protein levels are shown for APOA4 using sandwich immunoassay and expressed as mean intensity fluorescence (MIF).
(EPS)
